# Phase 2 Trial of Ultrahypofractionated Image-guided Partial Breast Irradiation Following Lumpectomy with Optional Oncoplastic Reconstruction for Early-stage Breast Cancer

**DOI:** 10.1016/j.adro.2025.101817

**Published:** 2025-06-22

**Authors:** Vani Gupta, Andrew T. Wong, Rachel Radigan, Michelle Sahagian, Sophia L. Fu, Austin Barney, Jeffrey Pettit, Rishi Shah, Shridevi Singh, Jana Deitch, Johnny Kao

**Affiliations:** a The New York Institute of Technology College of Osteopathic Medicine, Old Westbury, New York; b Summit Health, Florham Park, New Jersey; c Good Samaritan University Hospital, West Islip, New York; d St. Catherine of Siena Hospital, Smithtown, New York

## Abstract

**Purpose:**

As accelerated partial breast irradiation is gaining widespread acceptance for low-risk breast cancers treated with breast conservation, its role following oncoplastic surgery remains controversial.

**Methods and Materials:**

We performed a prospective phase 2 trial of women aged 50 and older who were estrogen receptor positive with stage 0 to 1 breast cancer measuring ≤3 cm following successful lumpectomy with optional oncoplastic reconstruction. Patients were treated on the Varian Edge radiosurgery system to a prescribed dose of 30 Gy in 5 fractions, and the primary endpoints were feasibility and safety. Patient-reported cosmesis was assessed using the Breast Cancer Treatment Outcome Scale validated instrument.

**Results:**

From 2018 to 2022, 50 patients with 52 tumors with a median age of 76 were enrolled, including 79% invasive breast cancer with 48% undergoing oncoplastic reconstruction. With a median follow-up of 47 months, long-term patient-reported cosmesis was excellent in 89% of patients and good in 11% of patients. All patients were locally controlled, but there were 2 ipsilateral breast events consisting of an intramammary lymph node failure and second primary triple-negative breast cancer outside the radiation field, both successfully salvaged with further local and systemic therapy.

**Conclusions:**

In carefully selected patients with low-risk early-stage breast cancer, patients treated with a 5-fraction regimen of partial breast irradiation achieve excellent cosmetic and oncological outcomes. Oncoplastic reconstruction was not a contraindication to partial breast irradiation.

## Introduction

For early-stage breast cancer, breast conserving therapy consisting of lumpectomy followed by adjuvant radiation therapy achieves long-term overall survival equivalent to mastectomy with a low rate of recurrence in the treated breast.^1^ Compared to mastectomy, patients undergoing breast conservation therapy have a more positive attitude about body image and improved physical and sexual function.^2^

Omitting 3 to 5 weeks of whole breast radiation therapy (WBRT) in low-risk breast cancer has been proposed but remains controversial.^3^^,^^4^ After breast conserving surgery, adjuvant radiation therapy improves local control and overall survival compared to lumpectomy alone.^5^ Even among highly selected older patients with predominantly luminal A subtype receiving hormone therapy, adjuvant radiation therapy reduces the 10-year relative risk of local failure by 80% to 90%.^4^^,^^6^ Traditionally administered once per day over 3 to 5 weeks, WBRT has long been a standard treatment for patients with breast cancer.^7^ In the absence of adjuvant radiation therapy, the vast majority of local recurrences occur in the region of the primary tumor.^8^

Very brief courses of partial breast irradiation have emerged as a highly promising and more effective alternative to radiation omission.^3^^,^^9^ In the absence of an extensive intraductal component, pathologic analysis of mastectomy specimens failed to detect malignant foci beyond 2 cm of the index lesion in the vast majority of patients.^8^ In a recent meta-analysis, accelerated partial breast irradiation (APBI) delivered via external beam radiation therapy or brachytherapy achieves equivalent rates of 10-year local control with less acute toxicity than WBRT.^10^ Three randomized trials have suggested improved long-term cosmesis with external beam partial breast irradiation to 30 to 40 Gy in 5 to 15 once daily fractions compared to WBRT to 40 to 50 Gy in 15 to 25 fractions.^11^, ^12^, ^13^ In contrast, twice daily external beam APBI to 38.5 Gy in 10 fractions resulted in worse cosmesis and intraoperative radiation results with a higher risk of local failure.^7^^,^^14^

Modern breast surgery increasingly includes oncoplastic reconstruction with the goal of further improving cosmesis while still maintaining oncologic outcomes.^15^ Oncoplastic surgery allows for surgical resection and local tissue rearrangement of breast parenchyma with the goal to create a more homogenous appearance of the breast after volume loss. Aesthetically satisfactory incision placements, including periareolar incisions, doughnut or crescent mastopexy incisions, inframammary incisions and axillary incisions, do not necessarily correlate with tumor location.^15^

The potential challenges of accurately targeting the resection cavity at risk after oncoplastic surgery for partial breast irradiation have been extensively reviewed.^16^^,^^17^ With shifting of the tumor bed secondary to oncoplastic surgery, even with predefined placement of clips, misalignment of the target volume can occur with radiation therapy planning.^16^ Despite these challenges, there is no convincing evidence that oncoplastic surgery compromises radiation efficacy. Given the emphasis of oncoplastic surgery on cosmesis, it makes logical sense to integrate partial breast irradiation because this technique appears to achieve improved cosmetic outcomes compared to whole breast radiation. Building on extensive institutional experience with catheter-based breast brachytherapy dating to 2005 and the pioneering work at the University of Florence, we developed a phase 2 trial of a 5-fraction regimen of partial breast intensity modulated radiation therapy (IMRT) using the Varian Edge radiosurgery system in patients undergoing oncoplastic reconstruction.^18^

## Methods and Materials

### Study design and participants

This single-arm phase 2 trial was approved by the Institutional Review Board, and all participants provided written informed consent. Eligible patients were women 50 years or older with unifocal stage 0 to 1 breast cancer measuring ≤3 cm undergoing breast conserving surgery with negative surgical margins (recommended ≥2 mm final margin width allowing for reexcision when indicated), estrogen receptor (ER) positive, human epidermal growth factor receptor 2 (HER2) negative, and no lymphovascular invasion. Patients with invasive breast cancer had a negative sentinel node (N0[i-]). Patients undergoing oncoplastic reconstruction were eligible, and placement of surgical clips or Biozorb was encouraged but not required. Oncoplastic reconstruction was classified by involvement of a plastic surgeon and level 1 or level 2 volume displacement or volume replacement through extensive review of operative reports.^15^

### Radiation treatment technique

Patients were simulated in the supine position using a breast board with the ipsilateral arm abducted and rotated to allow the breast to remain in a natural reproducible position.^1^ The tumor cavity was defined on computed tomography simulation by visible seroma, surgical clips, and surgical scar supplemented by presurgical imaging that included routine breast magnetic resonance imaging (MRI). Because breast MRI is performed in the prone position, whereas the patient was simulated supine, MRI information was incorporated via side-by-side cognitive fusion by the treating physician. The planning target volume (PTV) was defined as the tumor cavity + 1.5 cm cropped 5 mm off skin, cropped off chest wall, and treated to 26 to 30 Gy. For patients with low and high dose PTV targets, the PTV30 was defined as tumor cavity + 5 to 10 mm using a simultaneous integrated boost technique. IMRT was used to achieve optimal dose conformity while sparing the heart, lung, uninvolved breast, and skin, adhering to NSABP B39 organ at risk constraints. Patients were planned using Eclipse version 15.5.

Patients were treated using the Varian Edge radiosurgery system with 6 MV photons equipped with a 6-degree of freedom robotic couch, cone beam computed tomography, and high definition multileaf collimators. Real-time image guidance was accomplished using optical surface monitoring. Treatment was delivered with free breathing on nonconsecutive days.

### Endpoints

The primary endpoints were safety and feasibility. We evaluated patients for toxicity, recurrence and survival outcomes, and quality of life. Local failures were defined as recurrences in the index quadrant. Regional recurrence was defined as nodal recurrence. Disease-free survival was defined as any relapse of breast cancer or death from any cause. Acute toxicities were defined as occurring within 90 days of treatment, and late toxicities were defined as occurring after 90 days. Toxicities were graded using the Common Terminology Criteria for Adverse Events v5.0, and physician-rated cosmesis was assessed using the 4-category Harvard Breast Cosmesis Scale.

### Patient-reported outcomes

Patient-reported outcomes were assessed at baseline and during follow up visits using the Breast Cancer Treatment Outcome Scale (BCTOS) self-report questionnaire, which informs quality of life in addition to cosmesis with high reliability. The results were analyzed as the mean of average breast pain at baseline and follow-up visits. Cosmetic outcome score was the arithmetic mean of the eight items that assess cosmetic outcome, seven items that access functional status, and three items that reflect breast specific pain on the BCTOS.

### Statistical analysis

Survival was calculated from the date of study enrollment to last follow-up or death. Statistical analysis was performed using Stata version 13.1.

## Results

### Patient characteristics and radiation dosimetry

A total of 50 patients with 52 tumors (41 with invasive breast cancer, 11 with ductal carcinoma in situ, and 2 patients with bilateral tumors) provided consent to participate in the trial between November 2018 and July 2022. The median age was 75 (range, 51-89), the median tumor size was 7 mm, all patients were ER positive, and 25 tumor cavities (48%) underwent oncoplastic breast surgery.

Of 25 oncoplastic surgeries, 18 were level 1 reconstructions performed by the breast surgeon alone, 4 were level 1 reconstructions performed by the plastic surgeon, and 3 were level 2 reconstructions performed by the plastic surgeon. The 3 level 2 reconstructions included 2 circumvertical reconstructions and 1 reduction mammoplasty. For oncoplastic surgeries, 16 of 25 (64%) tumor cavities had either surgical clips or Biozorb placed compared to 11 of 27 (41%) standard lumpectomy cavities.

Baseline patient and treatment characteristics are shown in Table 1. The most common IMRT technique was the 2 dose level simultaneous integrated boost used in 41 (79%) tumor cavities while the remaining 11 (21%) tumor cavities were treated with a single PTV30. The median PTV26 to PTV28.5 volume was 241 cc, and the median PTV30 volume was 142 cc (Fig. 2a-b). The median ipsilateral breast volume was 1016 cc. The median heart dose was 32 cGy, with a median of 37 cGy for left-sided tumors and 16 cGy for right-sided tumors.

**Table 1:** Patient and tumor characteristics

Patient characteristics	Median or count (%)
Age, y	75 (range 51-89)
Tumor size, cm	0.7 (range 0.1-2.6)
≤1	38 (73%)
1.1-2	10 (19%)
>2	4 (8%)
PTV30 volume, cc	142 (range 43-408)
PTV26 to PTV28.5 volume, cc	241 (range 113-709)
Margin width	
>2 mm or negative reexcision	44 (85%)
Negative but <2 mm	4 (8%)
Negative width not specified	4 (8%)
Side	
Left breast	31 (60%)
Right breast	21 (40%)
Oncoplastic surgery	
Yes	25 (48%)
No	27 (52%)
Tumor grade	
Grade 1	17 (33%)
Grade 2	32 (61%)
Grade 3	3 (6%)
Invasive	
Yes	41 (79%)
No	11 (21%)
Adjuvant therapy	
Hormone therapy	39 (75%)
Chemotherapy	0 (0%)
No adjuvant therapy	13 (25%)
Estrogen receptor	
Positive	52 (100%)
Negative	0 (0%)
HER2/neu status	
Positive	0 (0%)
Negative	41 (79%)
DCIS	11 (21%)
Ki-67 (%)	15 (range 3-30)
≤13.25	14 (27%)
>13.25	20 (38%)
Unknown	18 (35%)
Baseline physician-rated cosmetic score	
Excellent	45 (87%)
Good	7 (13%)

*Abbreviations*: DCIS = ductal carcinoma in situ; PTV = planned tumor volume.

### Acute and late toxicity

Radiation was generally well tolerated. Acute and late toxicities are described in Table 2. Both acute grade 2 toxicities were breast seromas requiring aspiration. No patient developed late grade ≥2 toxicity. In terms of adherence to hormonal therapy, 25% of patients did not receive any hormone therapy, 10% discontinued treatment within 1 year because of side effects, and 6% required a change in endocrine agent because of toxicity.

**Table 2:** Physician-graded acute and late toxicities

Grade	Definition	Acute toxicity, ≤90 d	Late toxicity, >90 d
0	None	41 (79%)	50 (96%)
1	Mild	9 (17%)	2 (4%)
2	Moderate	2 (4%)	0 (0%)
3	Severe	0 (0%)	0 (0%)

### Patient and Physician Reported Outcomes

**Figure 1:**
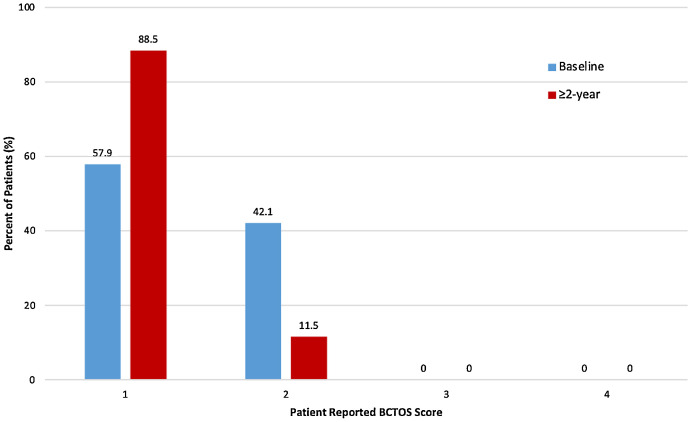
Percentage of 52 patients at each of the 4 cosmesis scores at baseline (blue) and greater than 2-year follow-up (red). *Abbreviation:* BCTOS = Breast Cancer Treatment Outcome Scale.

**Figure 2:**
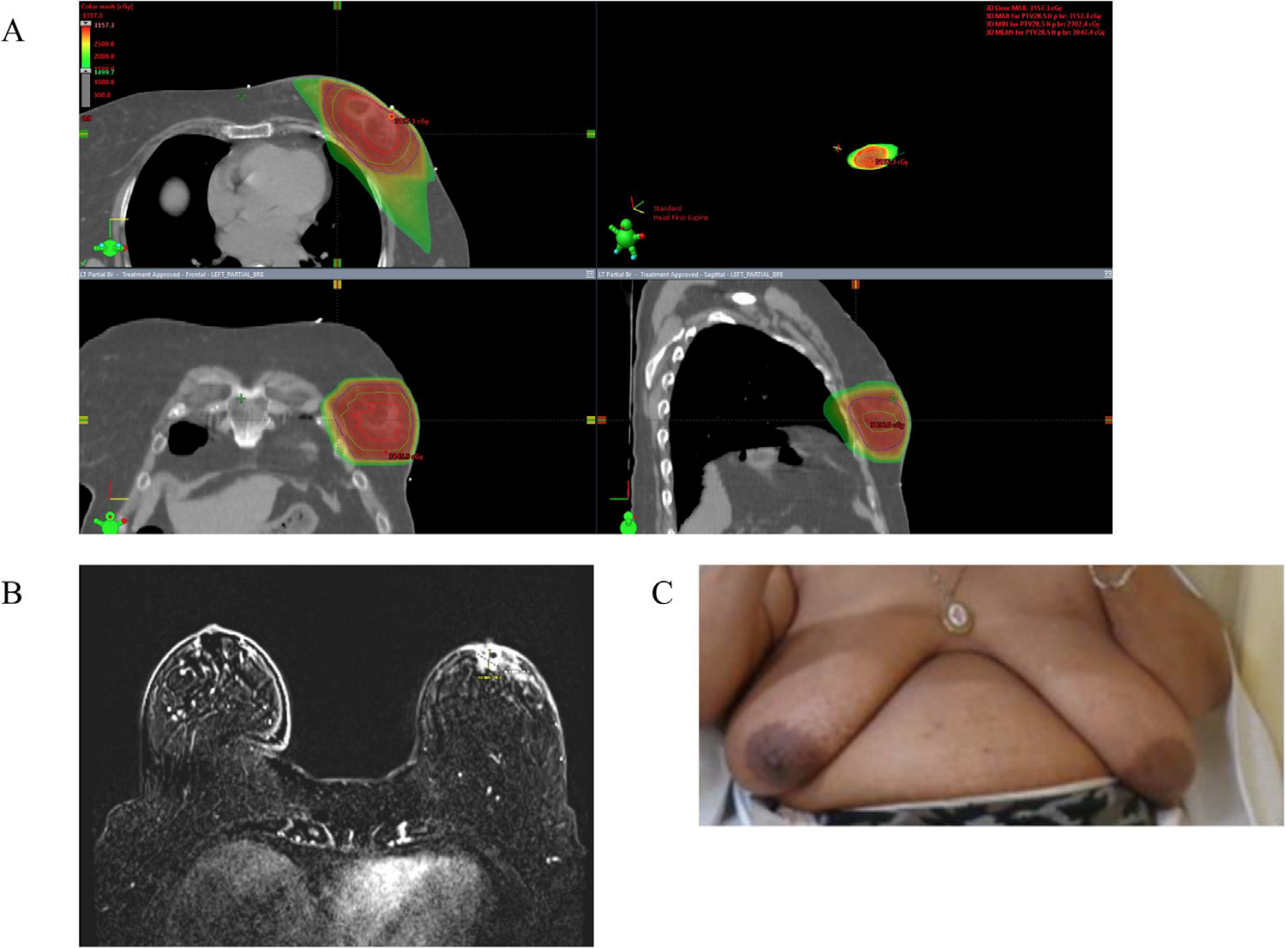
Radiation, MRI, and photographic imaging eliciting oncoplastic reconstruction. (A) Radiation dose distribution in a patient treated with lumpectomy with oncoplastic reconstruction. The tumor bed is contoured in red, the PTV30 in cyan, and the PTV28.5 in dark blue. (B) Bilateral breast MRI with contrast demonstrates subareolar enhancing mass to assist with target delineation despite oncoplastic reconstruction. (C) Photographic example of an excellent physician- and patient-reported cosmetic outcome despite a large right breast PTV30 volume of 397 cc. *Abbreviations:* MRI = magnetic resonance imaging; PTV = planned tumor volume.

Patient-reported outcomes were reported using BCTOS at baseline and follow-up visits. The overall average BCTOS scores at baseline were 58% excellent (91% standard lumpectomy, 13% oncoplastic), 42% good (9% standard lumpectomy, 88% oncoplastic), 0% fair to poor, and at ≥2 year follow-up, were 92% excellent (100% standard lumpectomy, 86% oncoplastic), 8% good (0% standard lumpectomy, 14% oncoplastic), and 0% fair to poor (Fig. 1; Fig. E1). The pre-radiation and long-term patient reported cosmesis, functional status and breast pain scores are displayed in Table 3. Cosmesis scores improved from baseline (47% none, 37% slight, 16% moderate, 0% large) to ≥2 year follow-up (73% none, 27% slight, 0% moderate to large). Late BCTOS cosmesis scores were better or unchanged compared to baseline for all patients. Functional status scores improve from baseline (68% none, 32% slight, 0% moderate to large) to ≥2 year follow-up (92% none, 8% slight, 0% moderate to large). Breast pain scores improve from baseline (58% none, 42% slight, 0% moderate to large) to ≥2 year follow-up (85% none, 15% slight, 0% moderate to large). BCTOS data were collected from 80% of patients while 20% of patients declined to participate. At last follow-up, physician-rated long-term cosmesis was rated as 72% excellent, 25% good, and 3% fair (Fig. 2c).

**Table 3:** Preradiation and long-term patient-reported cosmesis, functional status, and breast pain scores using the validated Breast Cancer Treatment Outcome Scale instrument

Grade	Definition	Baseline	3 mo	1 y	>2 y
Cosmesis					
1	None	47%	60%	83%	73%
2	Slight	37%	40%	8%	27%
3	Moderate	16%	0%	8%	0%
4	Large	0%	0%	0%	0%
Functional status					
1	None	68%	60%	92%	92%
2	Slight	32%	40%	8%	8%
3	Moderate	0%	0%	0%	0%
4	Large	0%	0%	0%	0%
Breast pain					
1	None	58%	47%	75%	85%
2	Slight	42%	40%	17%	15%
3	Moderate	0%	13%	8%	0%
4	Large	0%	0%	0%	0%

### Oncologic outcomes

**Figure 3:**
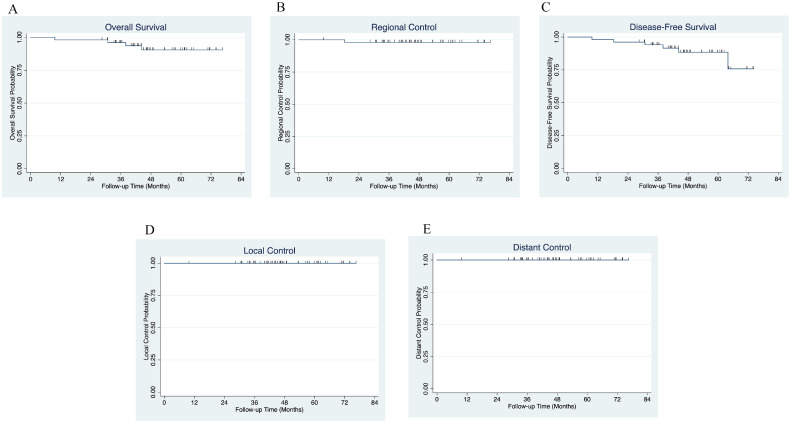
Kaplan-Meier curve eliciting 4-year oncological outcomes for patients treated with partial breast irradiation. (A) Overall survival (90%), (B) regional recurrence-free survival (98.%), (C) disease-free survival (88%), (D) local control (100%), (E) distant control (100%).

After a median follow-up duration of 47 months (range, 28-76) for surviving patients, the 4-year local control, regional control, distant control, disease-free survival, and overall survival were 100%, 98%, 100%, 88%, and 90%, respectively (Fig. 3).

There were 2 ipsilateral recurrences. Regional lymph node recurrence developed in a patient with high-grade invasive breast cancer who adamantly refused both whole breast irradiation and hormone therapy against medical advice. Although she was locally controlled, she developed intramammary and axillary nodal failure requiring repeat lumpectomy, axillary node surgery, and whole breast and regional nodal irradiation with eventual acceptance of hormone therapy. She remains alive and free of disease at last follow-up. An ipsilateral breast event developed in a complicated patient with a long-standing history of multiple contralateral breast cancers with biopsy-proven isolated supraclavicular lymph node recurrence in clinical remission. After partial breast irradiation, she was diagnosed with bone metastasis presumably related to her advanced contralateral breast cancer. At long-term follow-up, she was diagnosed with a fourth primary triple-negative breast cancer outside of the irradiated volume. She was salvaged with further surgery and systemic therapy. There were 3 deaths attributed to metastatic small cell lung cancer, endometrial cancer, and pancreatic cancer and a fourth death occurred at age 92 unrelated to breast cancer.

## Discussion

This trial confirms the safety and efficacy of APBI in well-selected patients undergoing either standard lumpectomy or mostly level 1 oncoplastic reconstructions at a community hospital. These data add to a rapidly growing body of literature of prospective trials investigating APBI delivered with a 5-fraction course of IMRT.^19^

The safe and effective delivery of partial breast irradiation on the Varian Edge radiosurgery system suggests that omitting fiducial marker placement without increasing margins does not compromise local control.^19^, ^20^, ^21^ Optical surface monitoring is a promising approach to noninvasively reduce setup uncertainty for a deformable target.^22^ Data from the FAST-FORWARD trial suggesting that whole breast doses as low as 26 Gy in 5 fractions are safe and effective raises the possibility of dose de-escalation below 30 Gy in the APBI setting.^23^ In the FAST-FORWARD trial, 27 Gy in 5 fractions to the entire breast was associated with worse cosmetic outcome than 40 Gy in 15 fractions.^23^ In our experience, most patients were treated to a PTV of cavity + 1.5 cm to 27 to 28.5 Gy with an integrated boost to cavity + 0.5 to 1 cm to 30 Gy. This represents a conservative approach to safely reduce dose compared to the standard Florence technique while maintaining excellent local control, treatment tolerance, and favorable cardiac dosimetry.

As the 5-fraction regimen of partial breast irradiation gains wider acceptance, the rationale for omitting radiation therapy continues to wane.^9^^,^^24^ In the PRIME II trial, omitting radiation therapy resulted in a 10.4 relative risk of ipsilateral breast recurrence despite ongoing hormone therapy.^9^ It bears mention that a relative risk reduction of >90% is exceedingly rare in clinical medicine or oncology.^25^ A recent systematic review identified 19 clinical trials that were terminated early because of efficacy, with unadjusted hazard ratios of 0.2 to 0.7.^26^ Although adjuvant breast radiation achieves a marked local control and overall survival advantage compared to lumpectomy alone on meta-analysis, researchers continue to pursue radiation omission trials with a noninferiority design despite the opportunity cost of diverting scarce clinical trial resources away from more productive research questions that seek to improve outcomes.^27^, ^28^, ^29^

After the publication of 10-year follow-up from the University of Florence trial, National Comprehensive Cancer Network guidelines now list 30 Gy in 5 fractions as the preferred approach to APBI.^12^ The University of Florence trial started in 2005, and there have been dramatic technological advances over the past 2 decades.^19^ Other groups have also developed modern methods using a variety of technological platforms to safely accomplish external breast APBI, confirming early but highly promising results.^20^^,^^21^^,^^30^^,^^31^ Although catheter-based brachytherapy has been extensively used in the past, enthusiasm for this technique has waned in recent years corresponding to the rise in oncoplastic surgery.^32^

For low-risk breast cancer, APBI is a safe and effective treatment approach, as it requires fewer treatment sessions and shorter overall treatment times along with favorable cosmetic outcomes with both conventional and oncoplastic lumpectomy. Although APBI following oncoplastic surgery remains controversial, we describe a method to overcome prior concerns of geographic miss by routinely using all available clinical and radiologic information while routinely employing a larger intermediate-risk PTV with a median volume of 241 cc.^16^ Until further data become available, the authors favor a larger APBI margin in the context of oncoplastic reconstruction that maintains a low mean heart dose of 0.3 Gy with excellent patient-reported BCTOS outcomes. Moreover, our center is a high-volume community hospital serving several breast surgeons and therefore contributes to the generalizability of APBI following oncoplastic surgery. The primary limitation of this trial is the small sample size, single institutional design, and incomplete compliance with patient-reported cosmesis data. Additional limitations of this study is that the vast majority of oncoplastic surgeries were type 1 volume displacement procedures, and our data are not broadly applicable to either type 2 volume displacement or volume replacement procedures. Our study was not designed to specifically comment on the additional value offered by incorporating presurgical MRI, surgical clips, or Biozorb or even wiring the surgical scar. Although still experimental, preoperative APBI is an alternative approach that eliminates the technical challenges associated with accurate contouring after oncoplastic reconstruction.^33^

## Conclusion

The modified Florence regimen delivered on the Varian Edge system appears safe and effective for appropriately selected patients with ER+/HER2− stage T0-T1N0[i-] breast cancer, even after oncoplastic surgery. Further investigation of this strategy is appropriate.

## Disclosures

The authors declare that they have no known competing financial interests or personal relationships that could have appeared to influence the work reported in this paper.

## Appendix Supplementary materials

APBI Supplemental Figure 1 Image, application 1

## Footnotes

Supplementary material associated with this article can be found in the online version at doi:10.1016/j.adro.2025.101817.
