# Supplementary figures and images for: Phase 2 Trial of Ultrahypofractionated Image-guided Partial Breast Irradiation Following Lumpectomy with Optional Oncoplastic Reconstruction for Early-stage Breast Cancer
Source: Adv Radiat Oncol. 2025 Jun 22;10(9):101817. doi: 10.1016/j.adro.2025.101817 (PMC12344782; doi:10.1016/j.adro.2025.101817)

## Slide 1
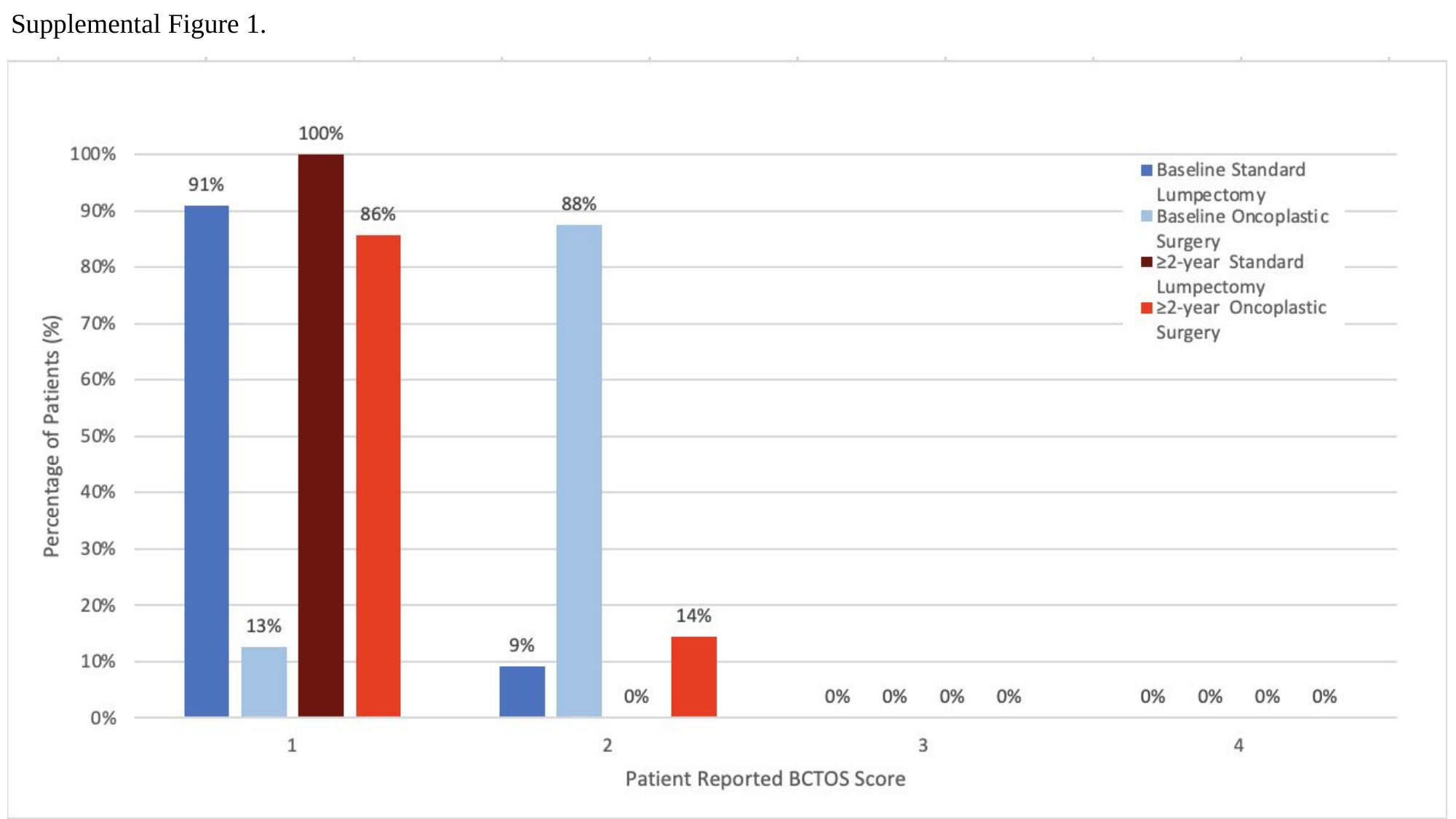

Supplemental Figure 1.

Supplement: APBI Supplemental Figure 1 [file mmc1.pptx]
